# Ulipristal Acetate Inhibits Progesterone Receptor Isoform A-Mediated Human Breast Cancer Proliferation and BCl_2_-L_1_ Expression

**DOI:** 10.1371/journal.pone.0140795

**Published:** 2015-10-16

**Authors:** Nathalie Esber, Florian Le Billan, Michèle Resche-Rigon, Hugues Loosfelt, Marc Lombès, Nathalie Chabbert-Buffet

**Affiliations:** 1 Institut National de la Santé et de la Recherche Médicale, Unité Mixte de Recherche-Scientifique 1185, Faculté de Médecine Paris Sud, Le Kremlin-Bicêtre, France; 2 Université Paris-Sud, Faculté de Médecine Paris Sud, Unité Mixte de Recherche-Scientifique 1185, Le Kremlin-Bicêtre, France; 3 HRA-Pharma, Paris, France; 4 Service d’Endocrinologie et des Maladies de la Reproduction, assistance Publique-Hôpitaux de Paris, Hôpital Bicêtre, Le Kremlin Bicêtre, France; 5 Service de Gynécologie Obstétrique Médecine de la Reproduction, Hôpitaux Universitaires Est Parisien site Tenon, AP-HP, Paris, France; 6 Institut National de la Santé et de la Recherche Médicale, Unité Mixte de Recherche-Scientifique 938, Centre de Recherche Saint Antoine, Université Pierre et Marie Curie, Paris, France; University of Wisconsin—Madison, UNITED STATES

## Abstract

The progesterone receptor (PR) with its isoforms and ligands are involved in breast tumorigenesis and prognosis. We aimed at analyzing the respective contribution of PR isoforms, PRA and PRB, in breast cancer cell proliferation in a new estrogen-independent cell based-model, allowing independent PR isoforms analysis. We used the bi-inducible human breast cancer cell system MDA-iPRAB. We studied the effects and molecular mechanisms of action of progesterone (P4) and ulipristal acetate (UPA), a new selective progesterone receptor modulator, alone or in combination. P4 significantly stimulated MDA-iPRA expressing cells proliferation. This was associated with P4-stimulated expression of the anti-apoptotic factor BCL_2_-L_1_ and enhanced recruitment of PRA, SRC-1 and RNA Pol II onto the +58 kb PR binding motif of the *BCL*_*2*_ *-L*_*1*_ gene. UPA decreased cell proliferation and repressed BCL_2-_L_1_ expression in the presence of PRA, correlating with PRA and SRC1 but not RNA Pol II recruitment. These results bring new information on the mechanism of action of PR ligands in controlling breast cancer cell proliferation through PRA in an estrogen independent model. Evaluation of PR isoforms ratio, as well as molecular signature studies based on PRA target genes could be proposed to facilitate personalized breast cancer therapy. In this context, UPA could be of interest in endocrine therapy. Further confirmation in the clinical setting is required.

## Introduction

Breast cancer, the most frequent cancer in women, is a hormone-dependent disease, with over 70% of sporadic breast tumors expressing estrogen and/or progesterone receptors (PR) [1]. Systemic anti-hormonal treatments used in clinical practice target the estrogen signaling pathway [2]. However, in the last decades, significant progress has been made in the understanding of the role of PR and its ligands in breast carcinogenesis [3–5].

Progesterone and progestins actions are mediated through their specific nuclear PR, with its two main isoforms PRA and PRB, in a tissue-specific, isoform-selective and ligand-dependent manner [3, 6]. Transcriptional activities of PRA and PRB isoforms are not similar, and both PR isoforms differentially regulate expression of a subset of target genes [7]. PRB functions as a strong transactivator and its transcriptional activity is down-regulated by the trans-dominant repressor PRA [8–9]. Progestin-induced cell spreading in ER-positive T47D cells expressing PR-A and PR-B isoforms was observed in cells overexpressing PRA by affecting cytoskeleton pathways and cell morphology [10]. Data obtained in MDA-MB 231 cells devoid of ER expression showed that PRA-transfected cells exhibited distinct morphological changes under antiprogestin ligands exposure as compared to PRB-transfected cells [11]. In this model co-transfection of ERα did not modify PRA *vs* PRB isoforms differences, suggesting an ERα- independent mechanism.

PR isoforms have an equimolar and coordinated expression in mammary epithelial cells under normal physiological conditions [12–13]. Dysregulation of the PRA/PRB ratio with high PRA expression levels in tumors leads to an impaired PR and ER signaling [12, 14]. Unbalanced PRA/B ratio has also been associated with tumor aggressiveness and poorer disease-free survival, and is observed in pre-neoplastic lesions from patients with breast cancer [3, 7, 12]. PRA is overexpressed as compared to PRB in 40% of ductal *in situ* carcinoma and invasive breast lesions [12]. Mutations in *BRCA1/2* genes are associated with PRA overexpression in tumor tissue [15–16] as well as in peritumoral normal breast tissue. Finally, recent data demonstrated in a large cohort that PRA overexpression is related to a shorter disease free survival in tamoxifen treated breast cancer patients [17]. These data suggest a direct role of PR in breast carcinogenesis with a differential contribution of PR isoforms.

New insights into the role of PR ligands in breast carcinogenesis have been highlighted in the past 20 years. Two large clinical studies have shown an increased risk of breast cancer in postmenopausal women treated with synthetic progestins combined to estrogens [18–19] as compared to women receiving estrogen-only treatment, suggesting a tumorigenic role of synthetic progestins.

Furthermore, clinical trials conducted in patients with metastatic, antiestrogen-resistant breast cancer, showed some efficacy of the antiprogestin mifepristone. In a preliminary study, administration of mifepristone decreased normal breast cell proliferation in women [20]. Finally, in a *BRCA1/P53* conditional breast knock-out mouse model, mifepristone was shown to prevent mammary tumorigenesis [15]. These data suggested that PR antagonists may contribute to control breast cancer development.

Ulipristal acetate is a recently released selective progesterone receptor modulator routinely used for emergency contraception [21] and mid to long term leiomyoma treatment [22–23]. This compound exerts mixed agonist/antagonist activities depending on the cellular context and has been shown to induce apoptosis in cultured leiomyoma cells [24] and in leiomyoma in vivo [25].

In a model of normal human breast cell culture, the selective progesterone receptor modulator (SPRM), ulipristal acetate (UPA), did not induce cell proliferation [26].

We used the newly established bi-inducible, basal breast cancer cell model, MDA-iPRAB, where ER expression is absent and PR is expressed independently of estrogens action [6, 27] to further evaluate the role of PR isoforms in breast carcinogenesis as well as the effects and mechanism of action of ulipristal acetate in breast cancer cells. We evaluated the effects of progesterone and UPA on cell proliferation, and the regulation of the anti-apoptotic marker BCL_2-_L_1_, as well as the molecular mechanisms involved. Overall, our findings propose that UPA induces a PRA mediated anti-proliferative effect and could be of interest in the management of human breast cancer patients.

## Materials and Methods

### Cell cultures and treatments

Bi-inducible MDA-iPRAB cell line was derived from human basal breast cancer MDA-MB-231 cell line (PR^-^, ERα^-^) (American Type Culture Collection, Manassas, VA, USA) as previously described [6]. Briefly, PRA and/or PRB expression could be induced at will by RSL1 (0.5 μM) (Exclusive Chemistry Ltd, Obninsk, Russia) and/or Dox (2 μM) (SigmaAldrich, Saint-Quentin Fallavier, France), respectively. Cells were grown in DMEM High Glucose with L-glutamine medium (Life Technologies, Saint-Aubin, France), 100 UI/mL penicillin, and 100 μg/mL streptomycin (GE Healthcare,Vélizy-Villacoublay, France) supplemented with 10% fetal calf serum (Biowest, Nuaille, France). For each experiment, cells were preincubated in steroid-free medium containing 2.5% dextran-coated charcoal treated serum (with DMSO or inducers) for at least 24 h before hormonal treatment. Ulipristal acetate (UPA) was from HRA Pharma (Paris, France). Progesterone and mifepristone (RU486) were from Sigma-Aldrich.

### Proliferation assays

Parental cells “clone 250” and MDA-iPRAB cells (iPRAB clone 38) (4,000 cells/ per well) were cultured in 96-well plates in steroid-free medium for 24 h. On day 0, 2, 4, RSL1 (0.5 μM) and/or Dox (2 μM) were added to fresh steroid-free medium to induce PRA and/or PRB expression, respectively. Cells were treated at the same time by Vehicle, P4 (1 nM) and/or UPA (1 μM) for 24 h. Cell proliferation assays were performed at day 1, 3, 5 by adding 20 μL of Cell Titer96 AqueousOne solution (Promega, Charbonnières-Les-Bains, France) into each well containing 100 μL of phenol red free culture medium (Life Technologies, Saint-Aubin, France). The plates were incubated at 37°C for 1 h and absorbance values were measured at 490 nm (A490) using a photometer (Victor 378, Perkin Elmer, Courtabœuf, France).

### Quantitative Real Time PCR

Total RNA was extracted from cells with TRI Reagent (Applied Biosystem) according to the manufacturer’s recommendations. One μg of total RNA was processed for reverse-transcribed-qPCR. Briefly, total RNA was treated with DNase I (Biolabs, Evry, France), then reverse-transcribed using the High-Capacity cDNA Reverse Transcription Kit (Life Technologies). cDNA were analyzed by quantitative RT-PCR using the Power SYBR Green PCR Master Mix (Life Technologies) with the indicated primers (300 nM, final concentration) (S1 Table) and a StepOne Real-Time PCR System (Life Technologies). Relative gene expression was calculated as a ratio of attomoles normalized by ribosomal rRNA 18S expression in femtomoles or by glyceraldehyde-3-phosphate-dehydrogenase (GAPDH) in attomoles.

### Immunoblotting

Cells were lysed in lysis buffer (150 mM NaCl, 50 mM Tris-HCl (pH 7.5), 5 mM EDTA, 30 mM Na pyrophosphate, 50 mM Na fluoride, 1% Triton X-100, protease and phosphatase inhibitor cocktails (Sigma) for 30 min on rotation at 4°C, followed by a centrifugation at 12,000 x *g* for 15 min at 4°C to clear debris. Samples were resolved by 7.5% sodium dodecyl sulfate gel electrophoresis and transferred onto nitrocellulose membranes. Antibodies used were anti-PR (NCL-L-PGR-312/2, Novocastra Laboratories, île Saint Martin, Nanterre, France), dilution 1:10,000; anti-Bcl-XL antibody (E18, ab32370, Abcam, France), dilution 1:1,000. Control antibodies: anti-α-tubulin antibody (Sigma) and anti-GAPDH (Sigma). Secondary antibodies: Goat anti-Mouse IgG (H+L) Cross Adsorbed Secondary Antibody (DyLight 800 conjugated, 680 conjugated) and Goat anti-Rabbit IgG (H+L) DyLight 800 conjugated, dilution 1:10,000 (Thermo Fisher Scientific, Rockford, IL, USA). These antibodies were diluted in Phosphate Buffered Saline and 0.1% Tween 20 buffer supplemented with 5% nonfat milk and added to the membranes for 1 h at room temperature or overnight at 4°C followed by incubation with the indicated secondary antibody for 1 h at room temperature. Target proteins were detected using Odyssey® Fc, Dual-Mode Western Imaging (Li-Cor, Lincoln, NE, USA) by Fluorescence and quantified.

### Chromatin Immunoprecipitation (ChIP)

MDA-iPRA cells were cultured for 36–48 h in DMEM/charcoal-stripped fetal bovine serum followed by 1 h treatment with P4 (10 nM) and/or UPA (1 μM). ChIP assays were performed as previously described [28] (#HighCellChIP kit, Diagenode, Seraing, Belgium) with 5 μg of the appropriate ChIP grade antibodies: Human anti-PR (Anti-PR, sc-7208, Santa Cruz Biotechnology, CA); Rabbit anti-SRC1 antibody (M-341, sc-8995,Santa Cruz Biotechnology), rabbit anti-Polymerase II antibody (H-224, sc-9001, Santa Cruz Biotechnology) and control unrelated antibody from the #HighCellChIP kit (Diagenode).

### Primer design for genomic amplification assays

Design of primer pairs containing PRbs (Progesterone receptor binding sites) was achieved with the NCBI software Primer BLAST. Human genome was used as database (hg19). Amplicon length was kept between 80 and 150 bp. Primers were from Eurogentec (Angers, France).

### Statistical Analysis

All data are mean ± SEM. Non-parametric Mann-Whitney statistical U-tests and ANOVA test (Kruskal-Wallis) were applied to determine all significant differences between experimental conditions, using the software Prism 5 (GraphPad Software, San Diego, CA). Statistical significances are indicated by * or x (1–3 symbols corresponding to P<0.05 or <0.01 or <0.001, respectively).

## Results

### PRA mediated MDA-iPRAB Cell Proliferation

**Fig 1:**
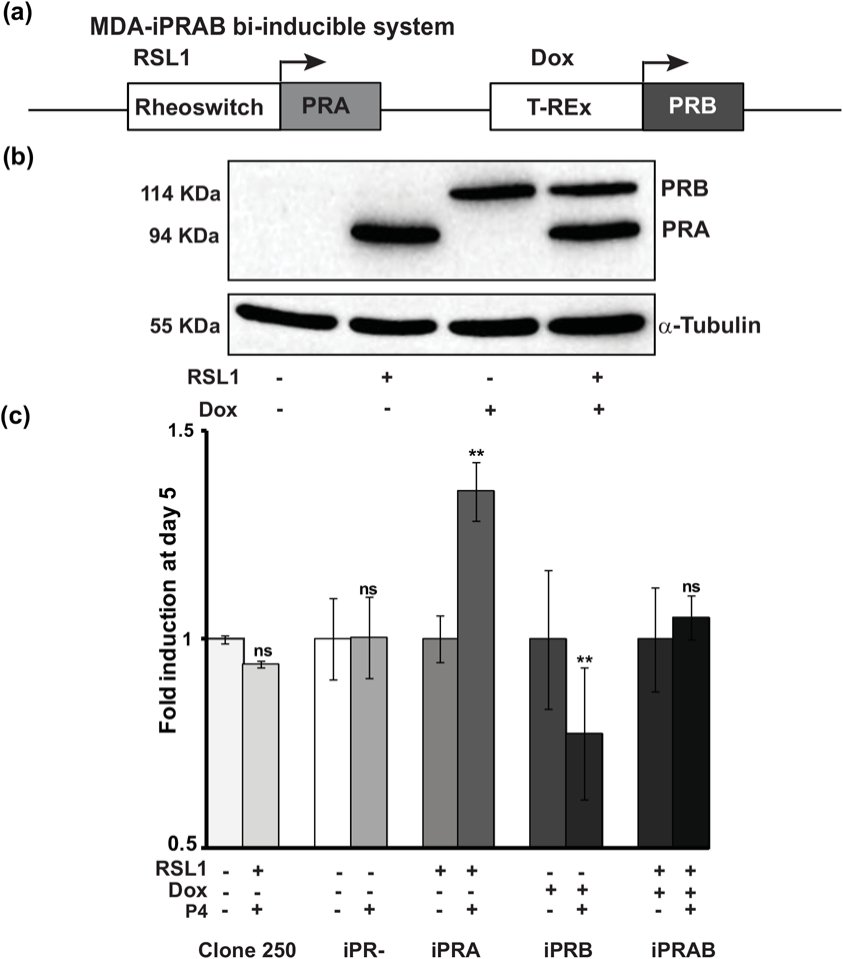
Progesterone-dependent activation of MDA-iPRA cell proliferation. **(a)** Schematic representation of the bi-inducible system inserted into the MDA-iPRAB cell line in which RSL1 and Dox selectively trigger expression of PRA and PRB isoform respectively, as previously described [6]. (**b**) PRA and/or PRB expression in MDA-iPRAB cell lines. MDA-iPRAB cells were incubated with DMSO or inducers, RheoSwitch Ligand (RSL1, 0.5 μM) and/or doxycycline (Dox, 2 μM) during 24 h. Western blot analysis of whole cell extracts was performed using anti-PR antibody recognizing both PR isoforms (Novocastra) or anti-tubulin antibody for sample loading control. **(c)** MDA-iPRAB cell proliferation assays were analyzed on day 1, 3 and 5. Data are expressed as fold induction compared to vehicle condition arbitrarily set at 1, and are means ± SEM from five independent cell cultures (n = 6 for each experiment. ** indicates p<0.01 compared to the vehicle-treated cells for each MDA-iPRAB cell line (non-parametric Mann Whitney t-tests).

We used the recently established bi-inducible MDA-iPRAB cell model to study the impact of PR isoform on human breast cancer cell proliferation [6]. This model results from genetically modified MDA-MB-231 human breast cancer cell line (PR^-^), generating various stable cell lines, MDA-iPR^-^, iPRA and/or iPRB cells. Two non-steroidal inducible systems, Rheoswitch and T-Rex were inserted into the same plasmid pZX-TR [6] (S1A Fig) and transfected into MDA-MB-231 cells generating parental and stable “clone 250” cell line. Subsequently, clone 250 cells were stably transfected with the bi-inducible system, pRheoswitch-PRA and pTRex-PRB, as schematically depicted on Fig 1A and S1B Fig. This bi-inducible system allows a bi-conditional expression of both PR isoforms, PRA at 94 kDa and/or PRB at 114 kDa, upon addition of RSL1 and/or Doxycycline (Dox) for 24 h, respectively (Fig 1B), while in the non-induced DMSO vehicle condition, no PR isoform expression was detected.

Given that the impact of P4 on cell proliferation was reported to be highly dependent on the cellular context [5, 29], we examined the influence of P4 on MDA-iPRAB cell proliferation. Unexpectedly, and unlike what we had published before with P4 at 10 nM, here we showed through repeated cell proliferation tests that P4 (1 nM) significantly increased MDA-iPRA cell proliferation at Day 5 (Fig 1C, S2A Fig). Of note, no effect of RSL1 and P4 was observed on cell proliferation of the parental clone 250, excluding a potential non-specific effect of these compounds (Fig 1C, left histograms). Moreover, P4 did not induce cell proliferation when none of the PR isoform was induced. In contrast, MDA-iPRB cell proliferation was significantly reduced in the presence of P4 (Fig 1C, right histograms), demonstrating that PRA specifically mediated cell proliferation in this model. Interestingly, co-expression of both PRA and PRB isoforms impeded P4-dependent MDA-iPRAB cell proliferation. These results are consistent with a PRA-mediated proliferative effect and PRB-mediated antiproliferative effect of P4 (Fig 1C).

### Anti-proliferative effect of UPA on MDA-iPRA cells

**Fig 2:**
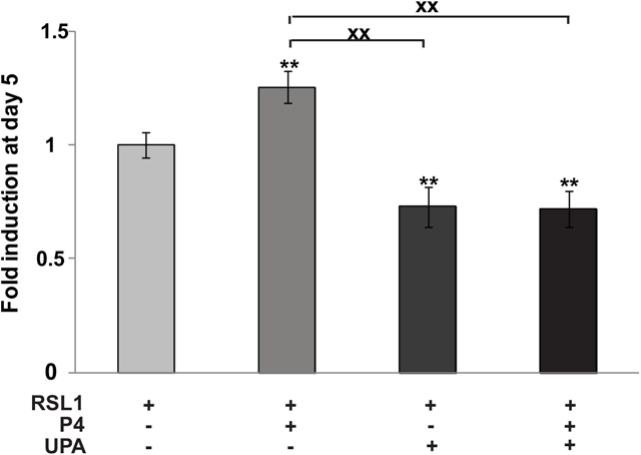
Ulipristal acetate (UPA) inhibits P4-dependent MDA-iPRA cell proliferation. MDA-iPRA cultured on 96-well plates, were treated with vehicle or P4 (1 nM) and/or UPA (1 μM) in fresh steroid-free medium containing DMSO or RSL1 (0.5 μM) on day 0, 2 and 4. Cell proliferation assays were performed using CellTiter96H AqueousOne Solution as described in Materials and Methods. The 490 nm absorbance was determined on day 1, 3 and 5. Data are expressed as fold induction compared to vehicle condition arbitrarily set at 1, and are means ± SEM from five independent cell cultures (n = 6 for each experiment). ** indicates p<0.01 compared to the vehicle-treated cells for each MDA-iPRA cell line while **xx** indicates p<0.01 compared to the P4-treated cells (non-parametric Mann Whitney t-tests).

We next examined the effect of the SPRM, UPA, on MDA-iPRA cell proliferation (Fig 2) and found that UPA at 1 μM alone or in association with P4 significantly inhibited cell proliferation as early as Day 3 (see also S2A Fig). This was consistent with our previous report showing that RU486 (Mifepristone) inhibits MDA-iPRA cell proliferation [6]. Of note, in the absence of PR, UPA has no effect, while UPA inhibited PRB-expressing cells and PRAB-expressing cells proliferation (S2B and S2C Fig). UPA has synergistic effects with P4 in MDA-iPRB cells.

Taken together, we reported the first cellular model in which P4 triggers PRA mediated human breast cancer cell proliferation *in vitro*. In this model, we unambiguously demonstrated that PRA isoform mediated P4-induced cell proliferation that was antagonized by UPA, highlighting the anti-proliferative role of UPA on human breast cancer cells.

### Key PRA target genes involved in human breast cancer cell proliferation: Effects of P4 and UPA

**Fig 3:**
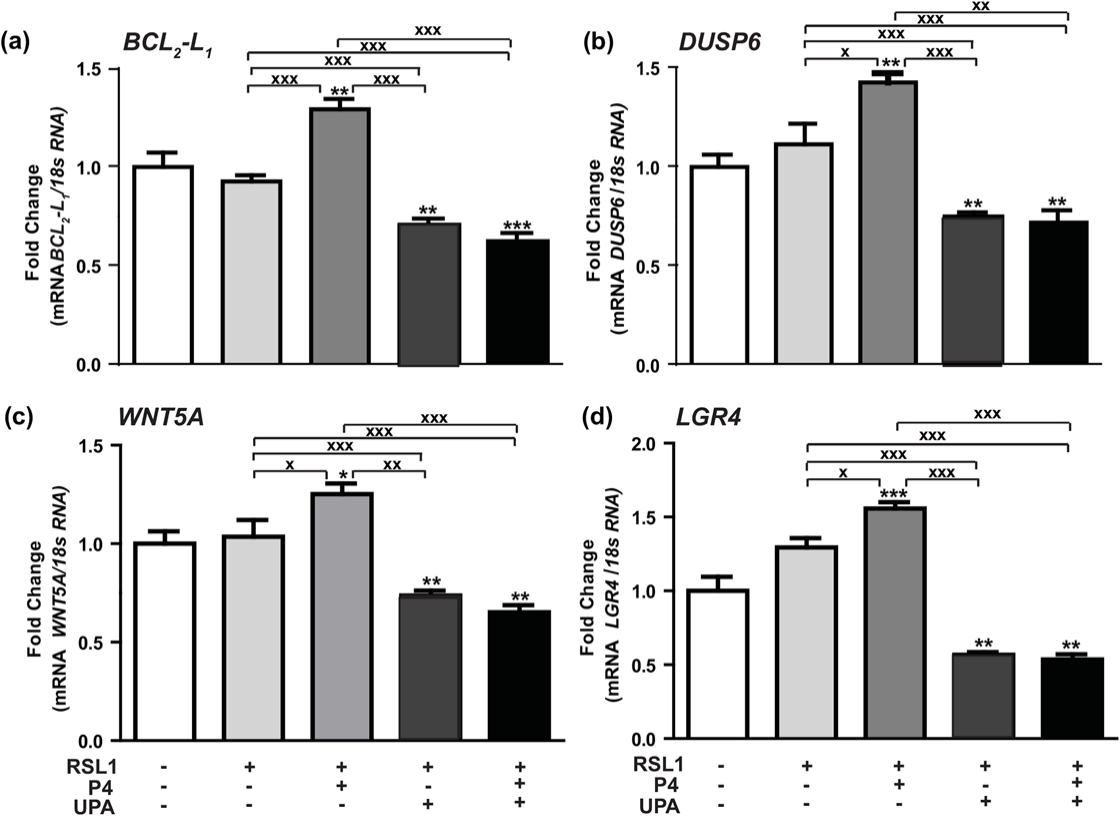
Hormonal regulation of P4-PRA-upregulated genes. (**a**) *BCL*_*2*_ *-L*_*1*_, (**b**) *DUSP6*, (**c**) *WNT5A*, (**d**) *LGR4* mRNA expression levels were determined in MDA-iPRA cells. Cells were treated for 6 h with vehicle, P4 (1 nM) and/or UPA (1 μM) in steroid-free medium, following 24 h induction of PRA expression using RSL1 (0.5 μM). RT-qPCR analyses were performed as described in Materials and Methods. Data are expressed as fold induction compared to vehicle condition arbitrarily set at 1, and are means ± SEM from three independent cell cultures measured in duplicate. *, **, *** symbols indicate p< 0.05, 0.01 and 0.001 respectively compared to the vehicle-treated MDA-iPR^-^ cells while **x, xx, xxx** symbols indicate p<0.05, 0.01 and 0.001 respectively compared to the V or P4-treated MDA-iPRA cells (non-parametric Mann Whitney t-tests).

**Fig 4:**
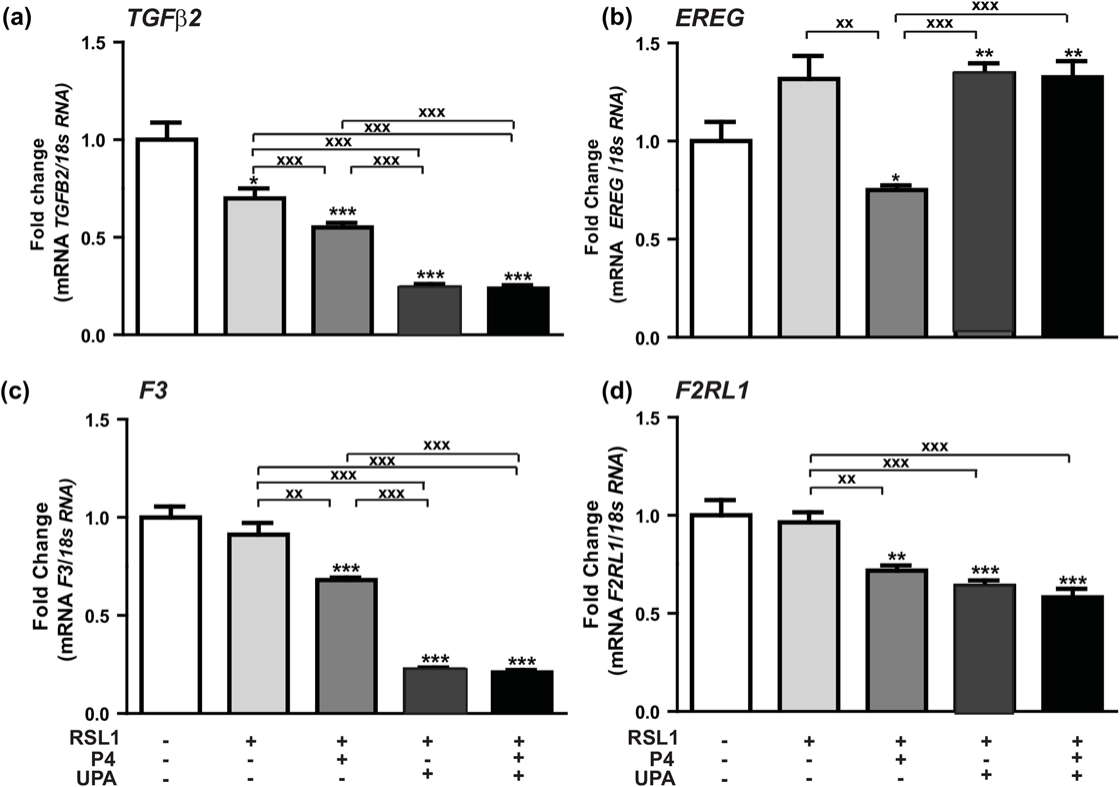
Hormonal regulation of P4-PRA-downregulated genes. (**a**) *TGFβ2*, (**b**) *EREG*, (**c**) *F3*, (**d)** *F2RL1*mRNA expression levels were in MDA-iPRA cells. Cells were treated for 6 h with vehicle, P4 (1 nM) and/or UPA (1 μM) in steroid-free medium, following 24 h induction of PRA expression using RSL1 (0.5 μM). RT-qPCR analyses were performed as described in Materials and Methods. Data are expressed as fold induction compared to vehicle condition arbitrarily set at 1, and are means ± SEM from three independent cell cultures measured in duplicate. *, **, *** symbols indicate p<0.05, 0.01 and 0.001 respectively compared to the vehicle-treated MDA-iPR^-^ cells while **x, xx, xxx** symbols indicate p< 0.05, 0.01 and 0.001 respectively compared to the V or P4-treated MDA-iPRA cells (non-parametric Mann Whitney t-tests).

We have previously identified by microarray analysis on the same MDA-iPRAB cell model, a subset of potential target gene candidates regulated in a P4-dependent and/or PR isoform specific manner [6]. Given the importance of PRA isoform, its impact on P4 signaling in mammary tumorigenesis processes such as cell growth, death and migration, and taking into account its role in mediating cell proliferation, we chose to focus our attention on some PRA-dependent upregulated (Table 1) or downregulated genes (Table 2) in the presence of P4. It would be thus possible to correlate P4-stimulated MDA-iPRA cell proliferation to the corresponding gene expression and to further evaluate the activity of the antiprogestin UPA in these processes. Among PRA-targeted genes transcriptionally stimulated (Fig 3) or inhibited by P4 (Fig 4), we examined the expression of few genes (*BCL*_*2*_ *-L*_*1*_, *DUSP6*, *WNT5A*, *LGR4*, *TGFβ2*, *EREG*, *F3*, *F2RL1)*, owing to their role in apoptosis, cell proliferation, metastasis, invasiveness, angiogenesis (Tables 1 and 2). We first validated the regulated expression of these genes by RT-qPCR analysis, after a 6 hr treatment by P4 following the same experimental duration as previously described [6]. We found that P4 significantly enhanced expression of *BCL*_*2*_ *-L*_*1*_ by 25% (Fig 3A), that of *DUSP6* by 50% (Fig 3B), that of *WNT5A* by 30% (Fig 3C) while *LGR4* expression was increased by 60% (Fig 3D). Interestingly, we demonstrated that the expression of these P4-upregulated genes was repressed by UPA alone or in combination with P4, confirming the antagonistic activity of UPA (Fig 3).

**Table 1:** Up-regulated PRA–target genes in the presence of P4.

*Class*	*Gene*	*Name*	FC	FI	Function
***A+&AB+***	***DUSP6***	***Dual specificity phosphatase 6***	**2**	**1.5**	**Transcriptional target of *P53* Interaction with PRB: proliferative transcriptional programs**
***A+&AB+***	***BCL***_***2***_ ***-L***_***1***_	***Bcl-2-like protein 1***	**1.5**	**1.2**	**Overexpression of Bcl-xL: loss of apoptosis**
***A+&AB+***	***TNFRSF11A***	***Receptor Activator of Nuclear Factor κ B (RANK)***	**1.9**	**1.6**	**Mammary cell proliferation, migration, cell invasion.**
***A+&AB+***	***WNT5A***	***Wingless-type MMTV integration site family*, *member 5A***	**1.8**	**1.3**	**Tumor suppressor gene Proliferation, differentiation, migration, adhesion, polarity**
***A+&AB+***	***LGR4***	***Leucine-rich repeat-containing G-protein coupled receptor 4 (GPR48)***	**1.8**	**1.6**	**Promoting cancer cell proliferation (Wnt signaling), Invasiveness and metastasis**
***A+&AB+***	***FOSB***	***FBJ murine osteosarcoma viral oncogene homolog B***	**1.8**	**1.5**	**Reduced FosB expression: dedifferentiation (breast tumorigenesis)**
***A+&AB+***	***TFPI2***	***Tissue factor pathway inhibitor 2***	**2.2**	**2.6**	**Low expression: breast cancer progression and poor outcome**
***A+&B+ & AB+***	***DUSP1***	***Dual specificity phosphatase 1***	**1.6**	**1.6**	**Cell cycle, dephosphorylation of MAP kinase. Antiproliferative actions of PR**

MDA-iPRAB cells were cultured during 24 h in steroid-free medium containing RSL1 (0.5 μM) and/or Dox (2 μM) to induce expression of PRA and/or PRB. Cells were treated by vehicle or P4 (1 nM) for 6 hours and a genome wide transcriptomic studies was performed as described in [6]. A total of 999 genes were affected by PR expression. These genes were separated into three groups: A and B and AB (co-expressed at similar levels relative to physiological conditions) regrouping genes that are regulated by either by PRA, PRB or PRAB. Tables above represent class *A+* genes regulated by PRA in the presence of the ligand; Class *A+&AB+*, genes responsive to liganded PRA and their expression may or may not be influenced by PRB co-expression; Class *A±&AB±* genes responsive to both liganded and unliganded PRA, and their expression may be influenced by PRB co-expression; Class *A+&B+& AB+*, genes that are commonly regulated by liganded PRA, PRB and PRAB. FC calculated either by microarray studies or by RT-qPCR analysis and their function in the cancer. FC: Transcriptional Fold Change calculated by microarray studies (FC*) and by RT-qPCR (FI, Fold Induction).

**Table 2:** Down-regulated PRA–target genes in the presence of P4.

*Class*	*Gene*	*Name*	FC	FI	Function
***A+***	***F2RL1***	***Coagulation factor II receptor-like 1***	**-1.9**	**0.7**	**Constitutive migration of MDA MB-231 Up regulation of VEGF**
***A+&AB+***	***EREG***	***Epiregulin***	**-1.7**	**0.7**	**Breast tumor progression *(*paracrine mechanism)**
***A+&AB+***	***F3***	***Coagulation factor III (tissue factor)***	**-2.4**	**0.7**	**Promigratory, invasive, proangiogenic phenotype**
***A+&AB+***	***TGFB2***	***Transforming growth factor-beta 2***	**-1.8**	**0.6**	**Breast cancer invasion Tumor suppressive (early stages) Malignant conversion and progression (later stages)**
***A+&AB+***	***ADRB2***	***Beta-2 adrenergic receptor***	**-1.7**	**0.6**	**Genetic variation and polymorphism: risk for breast cancer**
***A+&AB+***	***GJB2***	***Gap junction beta-2 protein*, *connexin 26***	**-1.8**	**0.5**	**Invasion of ductal breast carcinomas**

MDA-iPRAB cells were cultured during 24 h in steroid-free medium containing RSL1 (0.5 μM) and/or Dox (2 μM) to induce expression of PRA and/or PRB. Cells were treated by vehicle or P4 (1 nM) for 6 hours and a genome wide transcriptomic studies was performed as described in [6]. A total of 999 genes were affected by PR expression. These genes were separated into three groups: A and B and AB (co-expressed at similar levels relative to physiological conditions) regrouping genes that are regulated by either by PRA, PRB or PRAB. Tables above represent class *A+* genes regulated by PRA in the presence of the ligand; Class *A+&AB+*, genes responsive to liganded PRA and their expression may or may not be influenced by PRB co-expression; Class *A±&AB±* genes responsive to both liganded and unliganded PRA, and their expression may be influenced by PRB co-expression; Class *A+&B+& AB+*, genes that are commonly regulated by liganded PRA, PRB and PRAB. FC calculated either by microarray studies or by RT-qPCR analysis and their function in the cancer. FC: Transcriptional Fold Change calculated by microarray studies (FC*) and by RT-qPCR (FI, Fold Induction).

In contrast, P4 treatment significantly reduced the expression of *TGFβ2* by 60% (Fig 4A), and that of *EREG*, *F3* and *F2RL1* by 70% (Fig 4B, 4C and 4D) in MDA-iPRA cells, as revealed RT-qPCR analysis. Unexpectedly, UPA alone also inhibited the expression of *TGFβ2*, *F3* and *F*_*2*_ *RL*_*1*_ (Fig 4A, 4C and 4D) but not that of *EREG* (Fig 4B). However, the P4-dependent down-regulation of *EREG* expression was completely prevented by UPA (Fig 4B), consistent with a full PR antagonist activity of UPA. Of note, a strict correlation was observed between gene expression in transcriptomic and RT-qPCR analysis (Tables 1 and 2).

The expression of PRA-regulated genes was also analyzed in MDA-iPRB expressing cells. As shown in S3 and S4 Figs, expression of these genes did not vary in the presence of P4 and/or UPA treatment. Thus, we confirmed that *BCL*_*2*_ *-L*_*1*_, *DUSP6*, *WNT5A*, *LGR4*, *TGFβ2*, *EREG*, *F3*, *F2RL1* are indeed PRA specific genes and that UPA exerts an inhibitory effect by down regulating their expression only when PRA is expressed (MDA-iPRA).

Collectively, we demonstrated that the selected genes were specifically regulated by PRA isoform in the presence of its natural ligand P4. In addition, we also showed that UPA generally displayed PRA antagonist properties while, in some cases, UPA exerted a partial progesterone agonist activity consistent with the SPRM feature of his compound [11, 30].

### Regulation of BCL_2_-L_1_ protein expression by P4 and UPA in MDA-iPRA cells

**Fig 5:**
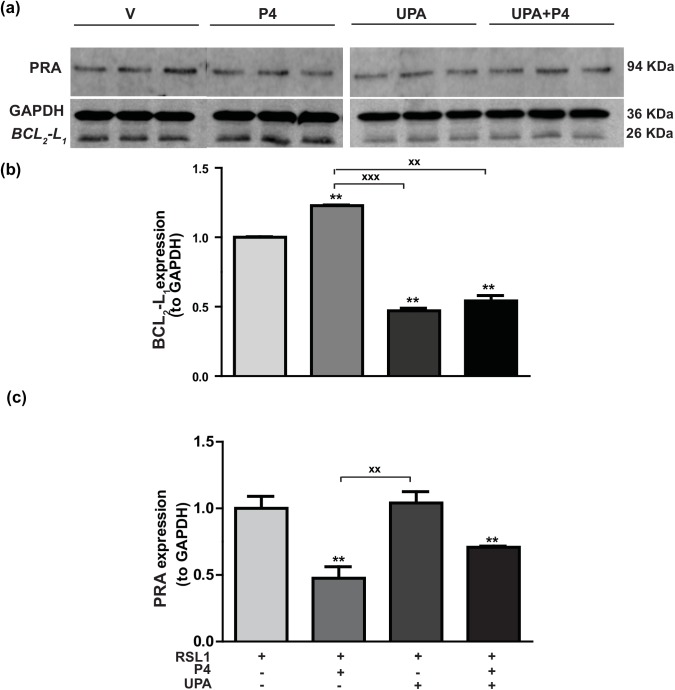
Hormonal regulation of BCL_2_-L_1_ protein expression in MDA-iPRA cells. Following 24 h induction of PRA expression using RSL1 (0.5 μM), MDA-iPRA cells were incubated in the presence or absence of P4 (1 nM) and/or UPA(1 μM) in steroid-free medium for 24 h. **(a)** Western blot analysis of whole cell extracts (35 μg Protein) loaded on a 12% acrylamide gel, membranes were incubated overnight with a 1:1,000 dilution of anti-BCL_2-_L_1_ antibody (anti-Bcl-XL antibody, E18, ab32370, Abcam, France), and an anti-PR antibody recognizing both PR isoforms (Novocastra) or anti-GAPDH antibody (G9545—anti-GAPDH antibody produced in rabbit). **(b)** Band intensities of BCL_2-_L_1_ (26 kDa) and GAPDH (36 kDa) used as a sample loading control were quantified and BCL_2-_L_1_/GAPDH ratio were determined and expressed in arbitrary units and normalized to vehicle. Relative BCL_2_L_1_ protein expression is presented. **(c)** Band intensities of PRA (94 kDa) and GAPDH (36 kDa) used as a sample loading control were quantified and PRA/GAPDH ratio were determined and expressed in arbitrary units and normalized to vehicle. Relative PRA expression is presented as well. Histograms are means ± SEM of three independent experiments. ** Symbol indicates p<0.01 compared to the vehicle-treated MDA-iPRA cells while **xx** symbol indicates p<0.01 compared to the P4-treated MDA-iPRA cells (non-parametric Mann Whitney t-tests).

Since P4 stimulated *BCL*_*2*_ *-L*_*1*_ mRNA levels (Fig 3A), we next examined the hormone regulation of the anti-apoptotic factor BCL_2_-L_1_, at the protein level. As illustrated in Fig 5A, the 26 kDa band corresponding to BCL_2_-L_1_ protein was significantly increased by 23% in MDA-iPRA cells upon 24 h exposure to P4 as revealed to quantification of band intensities relative to GAPDH (36 kDa) (Fig 5B). We also found that P4-stimulated BCL_2_-L_1_ expression was antagonized by UPA treatment which, when applied alone, significantly reduced by 2 fold factor the basal BCL_2_-L_1_ expression (Fig 5B). PRA expression was also quantified relative to loading control GAPDH (Fig 5C). As anticipated, PRA was significantly downregulated upon P4 exposure while UPA alone was ineffective in modulating PRA expression in MDA-iPRA cells. Taken together, we provide direct evidence of P4-stimulated MDA-iPRA cell proliferation, associated with BCL_2_-L_1_ expression, both being fully antagonized by UPA.

### PRA recruitment on *BCL*_*2*_ *-L*_*1*_ gene

**Fig 6:**
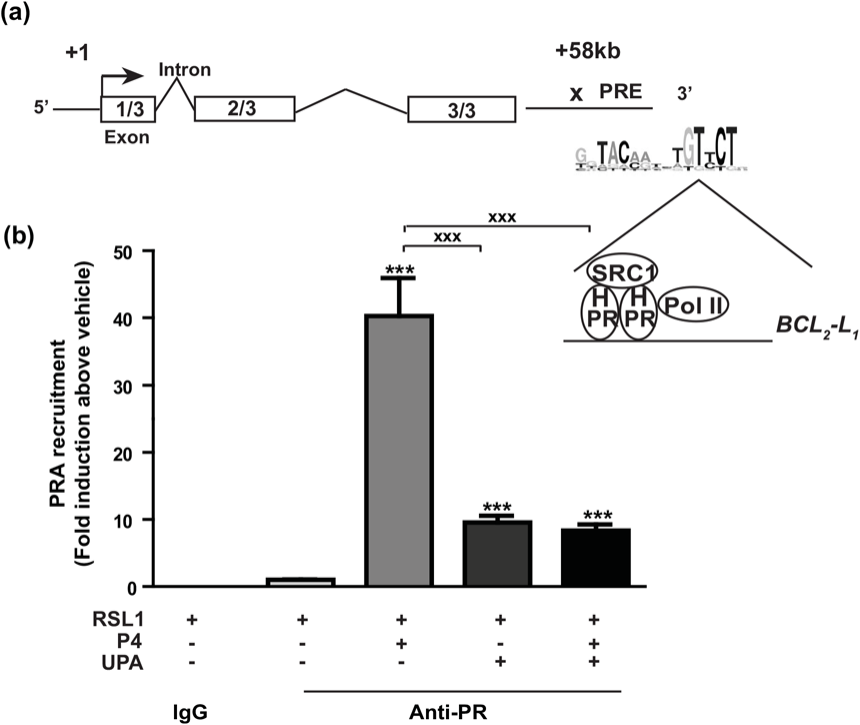
Progesterone enhances PRA recruitment on the intragenic region of *BCL*_*2*_ *-L*_*1*_ gene. **(a)** Schematic representation of *BCL*_*2*_ *-L*_*1*_ gene structure. It contains 3 exons. The genomic PR target motif previously identified [31] is located downstream the 3’end at position +58 kb of the transcription start site of *BCL*_*2*_ *-L*_*1*_ gene. The proposed PR response element (PRE) is illustrated on which the pre-initiation complex containing PR isoform, steroid receptor coactivator 1 (SRC1) as well as the RNA polymerase type 2 (Pol II) binds. **(b)** MDA-iPRA cells treated for 1 h with P4 (10 nM) and/or UPA (1 μM), were fixed and lysed and chromatin extracts subjected to ChIP assays using PR antibody (Anti-PR, sc-7208, Santa Cruz Biotechnology, CA) or unrelated rabbit IgG antibodies used as negative control. Immunoprecipitated and eluted DNA fragments were analyzed by real-time qPCR using primer pair encompassing genomic sequence of the +58 kb site of the *BCL*_*2*_ *-L*_*1*_ gene. Histograms represent the fold induction of PRA enrichment compared to vehicle condition arbitrary set at 1 and are means ± SEM of four independent experiments performed in triplicates. Statistical difference is indicated as compared to vehicle condition for PRA-induced cells treated by V (***, p<0.001), or by P4 (**xxx**, p<0.001) (Non-parametric Mann Whitney t-tests).

To better elucidate the molecular mechanisms by which P4-activated PRA regulated *BCL*_*2-*_ *L*_*1*_ gene expression in MDA-iPRA cells and based on previous studies performed in T47D cells [27], ChIP assays were performed to examine PRA recruitment on one genomic target motif, already established on *BCL*_*2*_ *-L*_*1*_ gene [31]. We chose the intragenic PR-binding sequence located at +58 kb downstream Transcription Start Site (TSS) of *BCL*_*2*_ *-L*_*1*_ gene as schematically depicted in the genomic structure of the human *BCL*_*2*_ *-L*_*1*_ gene (Fig 6A). P4 treatment induced a significant 40-fold increase in PRA recruitment on the well-documented +58 kb response element of the *BCL*_*2*_ *-L*_*1*_ gene, in comparison to the vehicle condition. Furthermore, UPA in combination with P4 markedly reduced P4-stimulated PRA enrichment on this PR-binding site (P< 0.001). Interesting enough, UPA alone was able to stimulate PRA recruitment by a 10-fold factor (Fig 6) as well as a substantial recruitment of the well-known SRC1 coactivator however to a lesser extent than that induced by P4 (2 *vs* 8 Fold) (S5A Fig). However, under these experimental conditions, as opposed to P4, UPA was unable to promote RNA Polymerase type II recruitment into the pre-initiation complex assembly (S5B Fig), required for transcriptional regulation. This finding is consistent with the UPA-dependent repression of *BCL*_*2*_ *-L*_*1*_ expression (see Figs 3 and 5).

## Discussion

We have exploited an original human breast cancer cell-based system in which cell proliferation is stimulated by progesterone through PR, using the recently established bi-inducible, ER negative, MDA-iPRAB cell line [6]. Several breast cancer cell models have been generated to study steroid effects, mostly focusing on the impact of estrogens and anti-estrogens [32–34]. The effects of progesterone and progestins on breast cancer cell proliferation *in vitro* drew contradictory results [35], although proliferative effects of progesterone have been reported in the ER^+^, PR^+^ T47D and MCF-7 breast cancer cells, without distinction between PR isoforms (review in [36]). Cell proliferation was evaluated in ER negative MDA-MB 231 cells transfected with each PR isoforms and cell proliferation inhibition by progesterone was observed in both cases [11]. In this report, progesterone was used at 0.1 μM and cells cultured in red phenol free DMEM supplemented with 5% charcoal treated fetal calf serum, while we used 2.5% FCS and 1 nM progesterone [11]. Progestin-stimulated cell proliferation has been reported to depend on progesterone concentration, mode of administration (continuous *vs* discontinuous) and may involve cross-talks with growth factors [36]. This may explain the discrepancy between our results and this study. We previously published that unliganded MDA-iPRA cells proliferate to a lesser extent than MDA-iPRB cells [6], in this study, we confirmed these findings (data not shown). We repeatedly and reproducibly observed that progesterone stimulated MDA-iPRA cell proliferation in 6 independent experiments (n = 6 per experiment). This is in accordance with PRA cells expressing increased responsiveness to stimulatory effects of progestins, as compared to cells expressing PRB reported by others [36–37].

One major advantage of our cell-based system is that one might selectively evaluate the effects of PR, its isoforms and ligands independently of estrogenic effects, since proliferation of this ER^-^ cell line is estrogen-independent. Moreover, the bi-inducible PR expression by non-steroid inducers is genomically stable, the non-induced cell line constituting a reliable negative control [6]. We cannot exclude that the absence of ERα (and estrogens) might modify the expression of co-regulators required for PR signaling and thus influencing transcriptional responses to PR isoforms. However data from previous reports did not show any influence of ERα transfection on PR ligands induced morphological changes in PR transfected MDA-MB 231 cells [11]. Taking into account these limitations, our studies provide new insights into the understanding of the functional consequences of imbalanced PRA/PRB ratio in the context of human breast cancer cells.

Interestingly, as opposed to the already known P4-biphasic regulation of cell proliferation observed in estrogen dependent T47D-YB cell line constitutively expressing PR-B [38], we report that P4 stimulates cell proliferation of bi-inducible MDA-iPRAB cells only when PRA isoform is expressed, while P4 exerts an anti-proliferative effect in PRB-expressing cells, without any biphasic effect. This underlines intrinsic differences in cell lines, in particular the MDA MB cell line is derived from a basal breast cancer [39] while the T47D cell line is derived from a luminal breast cancer [40].

In this model as well, PRA and PRB exhibit distinct transcriptional activities and regulate specific gene transcription. We have previously shown that PRA coexpression potentiated PRB-mediated cancer cell migration and that PR isoforms differentially regulated expression of major players of cell migration [41] such as urokinase plasminogen activator (uPA), its inhibitor plasminogen activator inhibitor type 1 (PAI-1), uPA receptor (uPAR), and β1-integrin, which affect focal adhesion kinase (FAK) signaling. PRA, as well as PRB, interacted with FAK complexes, whose degradation was coupled to a progestin-dependent turnover of PRA and PRB.

Our previous transcriptomic data allowed us to select PRA specific target genes [6]. Among PRA-modulated genes involved in breast tumorigenesis (Tables 1 and 2), we more specifically evaluated by quantitative RT-PCR and confirmed that *BCL*_*2*_ *-L*_*1*_, *DUSP6*, *WNT5A*, *LGR4* expression were upregulated in the presence of progesterone, and that *TGFβ2*, *EREG*, *F3*, *F2RL1* were down regulated in the presence of progesterone.

*BCL*_*2*_ *-L*_*1*_ is one of the most important regulators of programmed cell death. Deregulation of BCL_2_-L_1_ expression contributes to oncogenic transformation of normal cells [42–43]. In our MDA-iPRA model, P4 stimulates BCL_2_-L_1_ transcript and protein expression, while BCL_2_-L_1_ expression was not regulated by P4 in the MDA-iPRB cell line. Induction of anti-apoptotic factors of the Bcl family by progestins has been reported in endogenously PR-expressing T47D cells [35, 44], however the specific role of PR isoforms has not been directly evaluated.

*WNT5A* and *LGR4* regulate cell polarity, proliferation, survival and aberrant regulation often leads to pathological conditions including cancer [45]. In T47D cells, it has been demonstrated that PRB-induced transcriptional up-regulation of WNT1 leading to activation of epidermal growth factor receptor (EGFR) and c-Src and Erk1/2 [46]. Here, we report that PRA mediates the P4-induction and UPA-inhibition of the expression of both *WNT5A* and *LGR4*, suggesting an essential function of PRA-mediated, P4 triggered- breast cancer cell proliferation.

*DUSP6* (the dual specificity phosphatase 6, MKP3) is a negative regulator of the MAPK pathway [47]. Previous studies demonstrated a direct interaction between PRB and DUSP6 [47], our study confirms that *DUSP6* is a PRA-target gene up-regulated by P4 in MDA cells.

*TGFβ2* and *EREG* (Epiregulin) were shown to be down regulated by progesterone in the presence of PRA in our model [48–49]. This is in accordance with previous data suggesting that PR isoforms expression correlated negatively with other members of the same gene family associated with tumor aggressiveness such as *EREG* and *TGF alpha* [50].

Our results thus suggest a specific role of PRA in breast carcinogenesis, including modulation of regulatory factors of cell proliferation, invasion, apoptosis, and angiogenesis. This may constitute a trail for further molecular characterization of breast tumors, where the evaluation of a panel of specific PRA target genes could be used to further identify poor prognosis tumors. Indeed, breast cancer is now considered as a plural disease whose molecular signature analysis is routinely used to define personalized therapeutic schemes [51].

In addition, our data bring new insights in the isoform specific molecular mechanisms of action of PR and its ligands. It has been shown that over 60% of PR binding sites are located more than 10 kb from the transcription start site [52]. Here we showed that PRA is recruited to an intragenic PR-binding site (PRbs) located at +58 Kb downstream transcription start site of *BCL*_*2*_ *-L*_*1*_ and close to the 3’-end of the gene. This PRb was previously reported to serve as a highly active hormone-dependent binding site [31]. The canonical coactivator SRC1 and the Pol II were also recruited to this +58 Kb site, in a P4 enhanced way. To our knowledge, this is the first report of a direct evaluation of PRA interaction with molecular partners in PR^+^ basal breast cancer cells, while almost all previous PR microarrays studies were performed on luminal breast cancer cells [53]. This issue has been only addressed once [54], functional analyses were conducted in estrogen-dependent T47D cells, however ChIP experiments were conducted in non mammary HeLa cells and did not specifically evaluate PRA recruitment.

To evaluate the potential activity of PR antagonists on PRA-mediated cell proliferation, we used the recently marketed selective progesterone receptor modulator (SPRM), ulipristal acetate (UPA), a specific, highly selective PR antagonist with no agonist activity towards other steroid receptors [55], with mixed PR agonist/antagonist properties depending on the tissue [56]. Of interest, we showed that UPA inhibited MDA-iPRA cell proliferation. The anti-proliferative effect of UPA was restricted to PRA-expressing cells, independently of the presence of P4 while it exerts partial agonist activity on MDA-iPRB cells. ChIP experiments demonstrated that UPA partially inhibited PRA, SRC1 and Pol II recruitment on *BCL*_*2*_ *-L*_*1*_ PR-binding site in the presence of P4, while UPA alone weakly enhanced PRA and SRC1 recruitment only. This absence of Pol II recruitment in the presence of UPA correlated with the lack of UPA-induced PRA-mediated BCL_2_-L_1_expression, and is consistent with UPA inhibitory effect on both P4-induced BCL_2_-L_1_ transcript and protein. This may be correlated as well to the inhibition of cell proliferation in the presence of UPA, as already observed for other tumor types *in vivo* [24]. PRB interaction with SRC1 in the presence of a non-commercially available SPRM (asoprisnil) has been reported using transactivation assays in T47D cells [57]. To our knowledge this is the first report on the molecular mechanism of action of UPA.

Our data suggest that PRA expression is associated with increased human breast cancer cell proliferation, further stimulated by P4. We also show that UPA is able to decrease cell proliferation in this PRA expressing model. This effect of UPA includes basal as well as progesterone stimulated cell proliferation. Thus, our findings suggest that UPA might be of interest in the adjuvant treatment of breast cancer, more specifically in ER- PR+ tumors.

In sum, we propose that future characterization of breast cancer should include PR isoforms evaluation to better specify tumor phenotype, and/or its molecular signature independently of estrogen signaling. Most notably, this should evaluate PRA target genes to identify poor prognosis tumors, and potential candidates for SPRM adjuvant therapy. This obviously requires prior confirmation in the clinical setting.

## Supporting Information

S1 Fig Schematic representation of stably transfected plasmids inserted into the parental clone 250 and the bi-inducible system applied to generate MDA-iPRAB cells for the conditional PR isoform expression in MDA-MB-231 cells [6]. **(a)** The two regulatory proteins systems RheoSwitch and T-Rex were inserted in the same plasmid pZX-TR besides the zeocin resistance gene. **(b)** PRA and/or PRB expression was induced under the control of two promoters used to establish the secondary stable MDA-iPRAB cell line. Addition of RSL1 and/or Dox selectively induces PRA or PRB isoform, respectively. In their absence, no PR expression is induced.
(EPS)

S2 Fig P4 and UPA regulation of MDA-iPRA cell proliferation (a), MDA-iPRB cell proliferation (b) and MDA-iPRAB cell proliferation (c). Following 24 h induction of PRA expression using RSL1 (0.5 μM), and PRB expression using Dox (2 μM) cultured MDA-iPRA in 96-well plates, were treated with vehicle, P4 (1 nM) and/or UPA (1 μM) for 5 d. Cell proliferation assays were performed on day 0, 3 and 5 using CellTiter 96H AqueousOne Solution as described in Materials and Methods. Data are expressed as fold induction (a) and absorbances (b, c) compared to vehicle-treated MDA-iPRA cells (a) and vehicle-treated MDA-iPRB cells (b) and vehicle treated MDA-iPRAB cells (c) and are mean of 6 independent determinations. *, **, *** symbols indicate p<0.05, 0.01 and 0.001 respectively compared to the vehicle-treated MDA cells (non-parametric ANOVA test (Kruskal-Wallis).
(EPS)

S3 Fig Regulation of P4-dependent and PRA-selective upregulated genes (a) *BCL*_*2*_ *-L*_*1*_, (b) *DUSP6*, (c) *WNT5A*, (d) *LGR4* mRNA expression levels were determined in MDA-iPRB cells. Cells were treated for 6 h with vehicle, P4 (1 nM) and/or UPA (1 μM) in steroid-free medium, following 24 h induction of PRB expression using Dox (2 μM). RT-qPCR analyses were performed as described in Materials and Methods. Data are expressed as fold induction compared to vehicle condition arbitrarily set at 1, and are means ± SEM from three independent cell cultures measured in duplicate. *, symbol indicates p< 0.05 compared to the vehicle-treated MDA-iPRB cells while **x, xx, xxx** symbols indicate p< 0.05, 0.01 and 0.001 respectively compared to the V or P4-treated MDA-iPRB cells (non-parametric Mann Whitney t-tests).
(EPS)

S4 Fig Regulation of P4-dependent and PRA-selective downregulated genes (a) *TGFβ2*, (b) *EREG*, (c) *F3*, (d) *F2RL1* mRNA expression levels were determined in MDA-iPRB cells. Cells were treated for 6 h with vehicle, P4 (1 nM) and/or UPA (1 μM) in steroid-free medium, following 24 h induction of PRB expression using Dox (2μM). RT-qPCR analyses were performed as described in Materials and Methods. Data are expressed as fold induction compared to vehicle condition arbitrarily set at 1, and are means ± SEM from three independent cell cultures measured in duplicate. No statistical difference was detected.
(EPS)

S5 Fig SRC1 and Pol II recruitment to the *BCL*_*2*_ *-L*_*1*_ gene. MDA-iPRA cells treated for 1 h with P4 (10 nM) or UPA (1 μM), were fixed and lysed and chromatin extracts subjected to ChIP assays using SRC1 antibody (Rabbit anti-SRC1 antibody, M-341, sc-8995, Santa Cruz Biotechnology) or Pol II antibody (Rabbit anti-Polymerase II antibody, H-224, sc-9001, Santa Cruz Biotechnology). Immunoprecipitated and eluted DNA fragments were analyzed by real-time qPCR using primer pair encompassing genomic sequence of the +58 kb site of the *BCL*_*2*_ *L*_*1*_ gene. Histograms represent the fold induction of SRC1 **(a)** or Pol II **(b)** enrichment compared to vehicle condition arbitrary set at 1 and are means ± SEM of three independent determinations.
(EPS)

S6 Fig Regulation of *FKBP5*, a PRB-dependent gene. mRNA expression levels were determined in MDA-iPRB cells. Cells were treated for 6 h with vehicle, P4 (1 nM) and/or UPA (1 μM) in steroid-free medium, following 24 h induction of PRB expression using Dox (2μM). RT-qPCR analyses were performed as described in Materials and Methods. Data are expressed as fold change compared to vehicle condition arbitrarily set at 1, and are means ± SEM from three independent cell cultures measured in duplicate. *, **, *** symbols indicate p<0.05, 0.01 and 0.001 respectively compared to the vehicle-treated MDA-iPRB^-^ cells, while **x, xx, xxx** symbols indicate p< 0.05, 0.01 and 0.001 respectively compared to the V or P4-treated MDA-iPRB cells (non-parametric ANOVA test (Kruskal-Wallis).
(EPS)

S1 Table List of Primer sequences of genes. (DOCX)

## Abbreviations

P4: progesterone
PR: progesterone receptor
UPA: ulipristal acetate
