# Supplementary material for: Ulipristal Acetate Inhibits Progesterone Receptor Isoform A-Mediated Human Breast Cancer Proliferation and BCl2-L1 Expression
Source: PLoS One. 2015 Oct 16;10(10):e0140795. doi: 10.1371/journal.pone.0140795 (PMC4608808; doi:10.1371/journal.pone.0140795)
Supplement: S1 Table — (DOCX) [file pone.0140795.s007.docx]

**S1 Table: List of Primer sequences**

| **Gene** | **Accession Number** | **Forward primer (5'->3')** | **Reverse primer (3'->5')** |
| --- | --- | --- | --- |
| *ADRB2* | **NM_000024.5** | ***ATTGAGACCCTGTGCGTGAT*** | ***AAGGCCTGACACAATCCACA*** |
| *BCL2L1* | **NM_138578.1** | ***CTGGGTTCCCTTTCCTTCCA*** | ***CCTGGTCCTTGCATCTTTATCC*** |
| *BCL2L1 (ChIP)* | **NC_000020.11** | ***GACAGGGTTTTACTGGGTA*** | ***GTGTAGAGCAGAGCTCAGTA*** |
| *DUSP1* | **NM_004417.3** | ***GTGAAGCAGAGGCGAAGCAT*** | ***GGGATGGAGACGGGGAAGTT*** |
| *DUSP6* | **NM_001946.2** | ***TCACCCCCAATTTGCCGAA*** | ***TTCTTGCCCCGGGCTTCAT*** |
| *EREG* | **NM_001432.2** | ***TGCCTGGGTTTCCATCTTCT*** | ***TTGAGCCACACGTGGATTGT*** |
| *F2RL1* | **NM_005242.4** | ***ATGGGGCACTCCAGGAAGAA*** | ***AGAGCTGCTCAGGCAAAACA*** |
| *F3* | **NM_001993.4** | ***GCAGTGATTCCCTCCCGAACA*** | ***TGACCACAAATACCACAGCTCCA*** |
| *FOSB* | **NM_006732.2** | ***GAAGAGGAGAAGCGAAGGGT*** | ***CCACCAGCACAAACTCCAGA*** |
| *GJB2* | **NM_004004.5** | ***ACTGAGACCCCAGGCTGTTA*** | ***CCTCTGTGGAACCTGGCTTT*** |
| *LGR4* | **NM_018490.2** | ***TGAAGCCATTCGAGGGCTGA*** | ***GCTGTTGTCATCCAGCCACA*** |
| *TFPI2* | **NM_006528.3** | ***GCTCAGGAGCCAACAGGAAA*** | ***CAGCTCTGCGTGTACCTGTC*** |
| *TGFB2* | **NM_001135599.2** | ***CGAGAGGAGCGACGAAGAGT*** | ***GGACTGTCTGGAGCACAAGC*** |
| *TNFRSF11A* | **NM_003839.3** | ***TCAGTACACACACGGCAAACTTT*** | ***GGGTAGCACATATCTTCTGGAAATG*** |
| *WNT5A* | **NM_003392.4** | ***GCACCAGAGCAGACAACCTATTT*** | ***AAACGTGGCCAGCATCACAT*** |
